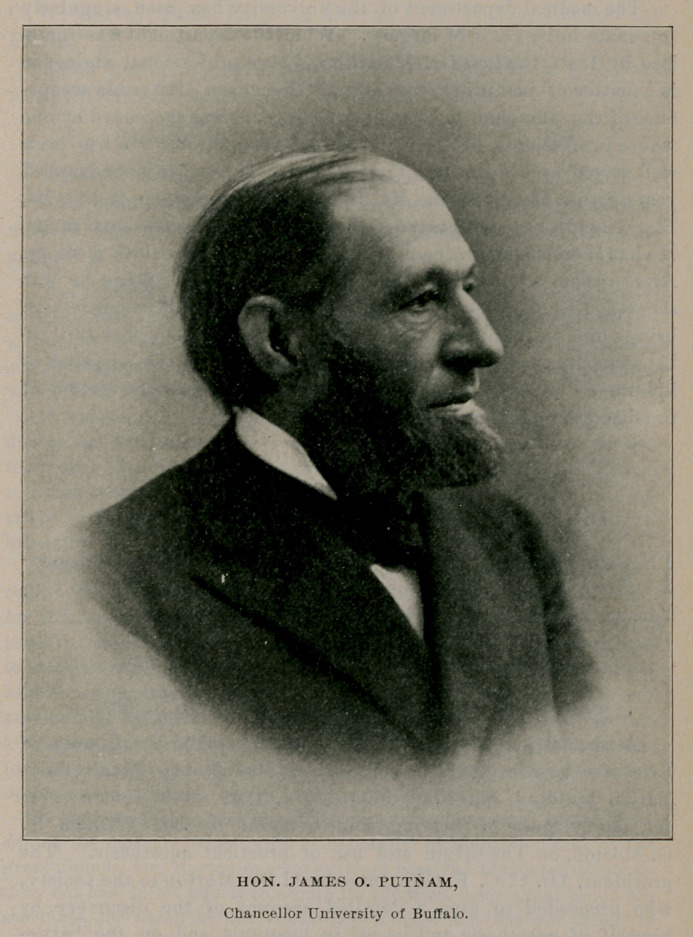# Introductory Remarks at the Semi-Centennial Commemoration of the Discovery of Anesthesia

**Published:** 1896-11

**Authors:** James O. Putam

**Affiliations:** Chancellor of the University


### INTRODUCTORY REMARKS AT THE SEMI-CENTENNIAL COMMEMORATION OF THE DISCOVERY OF ANESTHESIA.

By JAMES O. PUTNAM, Chancellor of the University.

"PEACE hath her victories." We meet on this occasion to commemorate a victory of science over pain. Important as have been the inventions of our century, which have given wings to commerce, which have put a girdle of instantaneous spoken thought around the globe, so bringing the great cities of the world into business and social unity ; inventions and discoveries which have wrested from nature the secret of her power, utilising it for the service of man, yet all these do not relieve him from physical suffering. But the discovery and application of anesthetics have conferred a blessing on our race no language can exaggerate. Imagine for a moment the carnage of a great battle in the wars of Napoleon, when the pain and anguish of those torn by shot and shell under the operations of the most skilful surgeons made the tens of thousands of battle-victims long for death more than for life; and then contrast that condition with the hospital service after one of the great battles of our late war, when by the temporary suspension of all sensibility following the application of anesthetics the most difficult operations of the surgeons are made painless, and the wounded soldier awakes to hope and gratitude. And what a boon the discovery brought to maternity, the greatest, perhaps, conferred since the first mother looked upon her first-born. The discovery has revolutionised surgery ; if it has not restored man to Paradise, it has delivered him from part of the primal curse.

Some years ago, wandering in the new public grounds of the City of Boston, I came upon a monument of granite and marble, surmounted by two figures representing the application of an anesthetic to a patient. Each side of the monument bore an inscription, all of kindred tenure. Two of them indicated the purpose of the monument, one in these words : "In gratitude for the relief of human suffering by the inhaling of ether, a citizen of Boston has erected this monument." The other seems to recognise the fulfillment on earth of the heavenly vision of St. John as recorded in the Revelations : "Neither shall there be any more

---

Delivered at the University of Buffalo, October 16, 1896.

pain." The name of the grateful donor, now known to be Thomas Lee, is too modestly withheld; it does not appear on the monument. At that time, 1868, there were two rival claimants to the honor of the discovery. I think we may here and in this presence take note of the fact that our century has emancipated—it is a century of emancipation—the medical profession in all its departments from imperfect systems which the ages had made venerable, but which courageous science has overleaped. To learn the causes of disease and to master it, to learn the laws of life and recast systems of the healing art in conformity to the law, is a passion of the best minds of the profession as intense as that which in other realms of science inspires the great discoverers and inventors. Is Edison greater in his sphere than was Pasteur in his? Napoleon drained two generations of Frenchmen of their best life-blood that he might float his ambition on a crimson sea. The fruitage of Pasteur's professional life is a blessing to the world which enrolls him with the greatest benefactors of the human race.

If it be desirable to have a sound mind in a sound body and to prolong human life so long as such conditions exist, and, I confess, without such conditions I think long life a questionable good; then, I think the studies of the medical profession in all its departments, to perfect the healing art, of more value to the human race than all the abstract speculations of philosophers and metaphysicians, which have no bearing on human well-being or on the practical relations of life.* We cannot live on mental gymnastics. Tennyson is right—

'Tis life whereof our nerves are scant,
O life, not death, for which we pant,
More life, and fuller that I want.

The Medical Department of the University of Buffalo honors itself in celebrating the semi-centennial of the first and successful application of anesthetics in surgery. It recognises the fact that the science of the profession is an ever-developing science and that its evolution follows in the line of sound education in its principles, and the rejection of every form of empiricism. The university is in sympathy with the demands of the state for advanced education and of increased terms of study before medical students shall take upon themselves the active responsibilities of the profession. The State Board of Regents is right in maintaining high standards in all the institutions under its charge. When asking

HON. JAMES O. PUTNAM,

Chancellor University of Buffalo.

for skill we will not accept quackery or ignorance. We would not die, even in a dentist's chair, from unskilful application of anesthetics, though we might, like fabled swans, die singing.

The medical department of the university has been singularly fortunate in its chair of surgery. When the department was organised in 1846, the late Dr. Hamilton, whose professional eminence is a matter of just pride, was called to that chair. After his acceptance of the same chair in New York College, he was succeeded by one whose professional life is one of proud distinction. He has been well styled by his brethren their Nestor. If now, in his advanced years, Prof. Moore be like Mt. Blanc, snow-capped, he is like Mt. Blanc in another respect, the highest peak in his professional range. He had a colleague in the late Dr. Miner, of cherished memory. After twenty-five years of service in our medical college he was succeeded by the present occupant of the chair, whose profession brings him daily into relations with anesthetics and whose skill in his specialty ranks him with the most eminent of the surgeons of his time. From both these gentlemen we shall hear the history of the discovery of anesthetics.

## Figures and Tables

**Figure f1:**